# Supplementary figures and images for: A-to-I RNA editing of CYP18A1 mediates transgenerational wing dimorphism in aphids
Source: eLife. 2025 Apr 3;13:RP96540. doi: 10.7554/eLife.96540 (PMC11968105; doi:10.7554/eLife.96540)

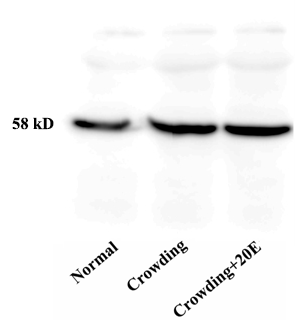

Supplement: Figure 3—source data 1. [file elife-96540-fig3-data1.zip › Fig. 3-source data 2.tif]

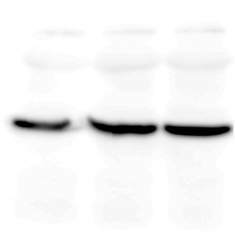

Supplement: Figure 3—source data 2. [file elife-96540-fig3-data2.zip › Fig. 3-source data 3.tif]

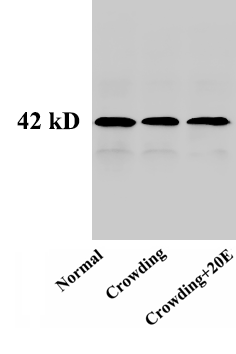

Supplement: Figure 3—source data 3. [file elife-96540-fig3-data3.zip › Fig. 3-source data 4.tif]

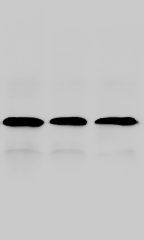

Supplement: Figure 3—source data 4. [file elife-96540-fig3-data4.zip › Fig. 3-source data 5.tif]

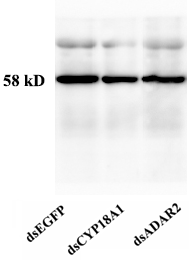

Supplement: Figure 4—source data 1. [file elife-96540-fig4-data1.zip › Fig. 4-source data 1.tif]

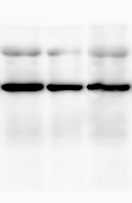

Supplement: Figure 4—source data 2. [file elife-96540-fig4-data2.zip › Fig. 4-source data 2.tif]

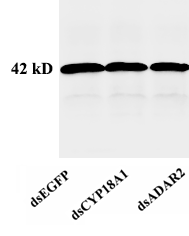

Supplement: Figure 4—source data 3. [file elife-96540-fig4-data3.zip › Fig. 4-source data 3.tif]

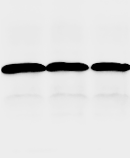

Supplement: Figure 4—source data 4. [file elife-96540-fig4-data4.zip › Fig. 4-source data 4.tif]

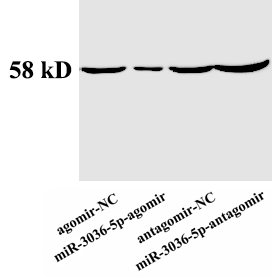

Supplement: Figure 6—source data 1. [file elife-96540-fig6-data1.zip › Fig. 6-source data 1.tif]

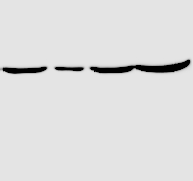

Supplement: Figure 6—source data 2. [file elife-96540-fig6-data2.zip › Fig. 6-source data 2.tif]

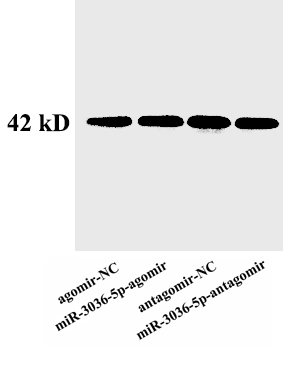

Supplement: Figure 6—source data 3. [file elife-96540-fig6-data3.zip › Fig. 6-source data 3.tif]

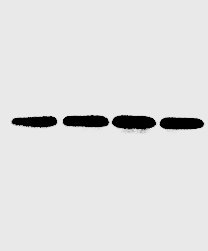

Supplement: Figure 6—source data 4. [file elife-96540-fig6-data4.zip › Fig. 6-source data 4.tif]

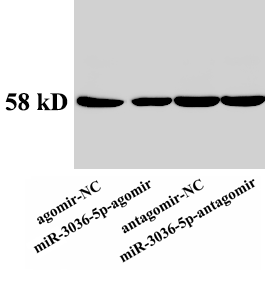

Supplement: Figure 6—source data 5. [file elife-96540-fig6-data5.zip › Fig. 6-source data 5.tif]

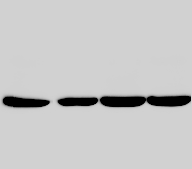

Supplement: Figure 6—source data 6. [file elife-96540-fig6-data6.zip › Fig. 6-source data 6.tif]

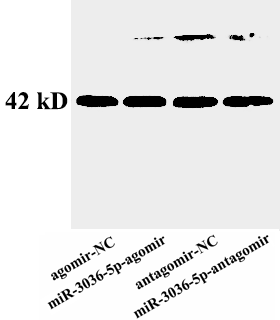

Supplement: Figure 6—source data 7. [file elife-96540-fig6-data7.zip › Fig. 6-source data 7.tif]

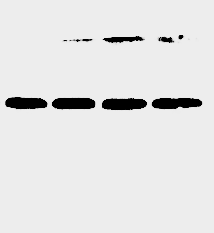

Supplement: Figure 6—source data 8. [file elife-96540-fig6-data8.zip › Fig. 6-source data 8.tif]
